# Extravascular lung water and pulmonary arterial wedge pressure for fluid management in patients with acute respiratory distress syndrome

**DOI:** 10.1186/2049-6958-9-3

**Published:** 2014-01-16

**Authors:** Wei Hu, Chang-Wen Lin, Bing-Wei Liu, Wei-Hang Hu, Ying Zhu

**Affiliations:** 1 Department of Intensive Care Unit, the First People’s Hospital of Hangzhou, Nanjing Medical University, Hangzhou 310006, China

**Keywords:** Acute respiratory distress syndrome, Extravascular lung water, Fluid management, Pulmonary artery wedge pressure

## Abstract

**Background:**

Extravascular lung water (EVLW) is a sensitive prognostic indicator of pulmonary edema. Thus, EVLW may be an advantageous method of fluid management. This study aims to evaluate the outcomes of using EVLW and pulmonary artery wedge pressure (PAWP) as strategies for fluid management in patients with acute respiratory distress syndrome (ARDS).

**Methods:**

Twenty-nine patients were randomly divided into the EVLW and PAWP groups. The survival rate, ICU (Intensive Care Unit) length of stay, duration of mechanical ventilation, acute lung injury scores, and oxygenation index of the EVLW and PAWP groups were compared.

**Results:**

No significant difference in the survival rates at 28 and 60 days (d) after treatment was found between the two groups (p = 0.542). The duration of mechanical ventilation and ICU length of stay were significantly lower (p < 0.05) in the EVLW group than in the PAWP group. The 7 d cumulative fluid balance was -783 ± 391 ml in the EVLW group and -256 ± 514 ml in the PAWP group (p < 0.05). Compared with the PAWP group, the EVLW group showed improved oxygenation index (p = 0.006).

**Conclusions:**

EVLW for fluid management improved clinical results in patients with ARDS better than PAWP.

## Background

Acute respiratory distress syndrome (ARDS) is a life-threatening form of lung injury that usually occurs with diseases, such as severe infection, multiple trauma, and burns. The condition occurs because of the diffuse pulmonary interstitial and alveolar edemas caused by the injury of pulmonary capillary and alveolar epithelial cells. When blood cells are low in oxygen, ARDS becomes a progressive disease that causes respiratory distress with extremely high mortality risk. Thus, ARDS is a serious threat that affects the quality of patients’ lives. Increased capillary permeability is a pathophysiological hallmark of ARDS, and the severity of pulmonary edema is positively associated with the prognosis of ARDS [1]. Therefore, active fluid management is clinically important to ameliorate pulmonary edema. However, fluid management in ARDS patients is difficult because a sufficient volume must be supplied to resuscitate the patient while avoiding the occurrence or exacerbation of pulmonary edema [2]. Pulmonary arterial wedge pressure (PAWP) and central venous pressure (CVP) have long been used for clinical fluid management in ARDS patients. However, these methods are not accurate because the relationship among PAWP, CVP, and cardiac preload may be affected by several factors [3,4]. The pulse-indicating continuous heart displacement monitoring technology (pulse-induced contour cardiac output; PiCCO) was applied. Extravascular lung water (EVLW) has also been used as a sensitive prognostic indicator of pulmonary edema. EVLW has become an important hemodynamic parameter for bedside monitoring [5-7]. Thus, EVLW may be a more advantageous method of fluid management [7-9]. In this study, we evaluated and compared the outcomes of ARDS patients treated with fluid management by EVLW and PAWP.

### Subjects and methods

#### Patients

This study was approved by the Ethics Committee. All 29 adult ARDS patients or their guardians signed an informed consent form upon recruitment and enrolment for this study. All patients were hospitalized at the ICU (Intensive Care Unit). Patients were diagnosed according to the American-European Consensus definition of ARDS provided in 1994: (a) acute onset; (b) bilateral infiltrates on chest radiograph consistent with pulmonary edema; (c) ratio of partial pressure of arterial oxygen (PaO_2_) to fraction of inspired oxygen (FiO_2_) less than 200; and (d) absence of left atrial hypertension. The study period lasted from January 2009 to November 2011. The study excluded patients with severe pulmonary hypertension, complete left bundle-branch block, pneumonectomy, interstitial lung disease, and bacterial endocarditis. Patients discharged against medical advice were also excluded.

The patients were randomly divided into the EVLW (*n* = 15) and PAWP (*n* = 14) groups. The causes of ARDS in the EVLW group included severe pneumonia (*n* = 6), abdominal infection (*n* = 3), multiple trauma (*n* = 2), H_1_N_1_ (*n* = 2), severe acute pancreatitis (*n* = 1), and irritant gas inhalation (*n* = 1). The causes of ARDS in the PAWP group included severe pneumonia (*n* = 5), abdominal infection (*n* = 3), multiple trauma (*n* = 1), H_1_N_1_ (*n* = 2), severe acute pancreatitis (*n* = 2), and urinary tract infection (*n* = 1). The general characteristics of the patients from the two groups are listed in Table 1. No significant differences were observed between the two groups (p > 0.05).

**Table 1:** The general characteristics of patients enrolled in the two groups

**Groups**	**n (cases)**	**Gender (cases)**		**Age (years)**	**APACHEα score**	**SOFA score**	**ALI score at baseline**	**Oxygenation index at baseline (mmHg)**
		**M**	**F**					
EVLW Group	15	8	7	50 ± 13	22.20 ± 3.14	9.33 ± 2.41	2.78 ± 0.45	127.57 ± 18.59
PAPW Group	14	9	5	49 ± 12	21.93 ± 3.77	9.36 ± 2.34	2.83 ± 0.53	133.73 ± 22.92

Data are shown as mean ± SD.

## Methods

All patients admitted to the ICU were treated by mechanical ventilation to manage the primary disease. Mechanical ventilation was performed according to the protective ventilation strategy recommended by the ARDS Network. In general, a pressure control ventilation or biphasic positive airway pressure mode was adopted, with the controlled peak airway pressure < 35 cm H_2_O, the plateau pressure < 30 cm H_2_O, and the force inspiratory oxygen and positive pressure respiration set according to titration. The patients were evaluated using Acute Physiology and Chronic Health Evaluation II and Sequential Organ Failure Assessment scores. For the EVLW group, a standard central venous catheter (Arrow Electronics, New York, USA) was inserted into either the internal jugular or subclavian location, and an arterial catheter (Pulsion Medical Systems, Munich, Germany) was inserted into the descending aorta via the femoral artery using the Seldinger technique. During fluid management, EVLW was measured every 8 h and maintained within 3 ml/kg to 7 ml/kg. When EVLW reached or exceeded 7 ml/kg, the patient was treated with diuretics or continuous renal replacement therapy (CRRT). For the PAWP group, a Swan-Ganz floating catheter (Arrow Electronics, New York, USA) was inserted into the pulmonary artery using the Seldinger technique. PAWP was measured every 8 h and maintained within 8 mm Hg to 12 mm Hg. When PAWP reached or exceeded 12 mm Hg, treatment with diuretics or CRRT was performed. All decisions on patient management, including fluid and ventilator management, were made by the primary intensive care physicians caring for the patient. This study was conducted in accordance with the declaration of Helsinki. The experiments were approved by the Ethics Committee of the first People’s Hospital. Written informed consent was obtained from all participants.

### Outcome variables

Vital signs, such as blood pressure, were recorded every hour. The 24 h intake and discharge fluid volume were also recorded daily. The acute lung injury score and oxygenation index were calculated every 24 h. Survival rates at 28 and 60 d after treatment were taken as the primary endpoints. Finally, the ICU length of stay (ICU LOS) was determined as a secondary endpoint to evaluate the curative effect.

### Statistical analysis

Statistical analysis was performed using the software program SPSS (version 18.0; Chicago, IL, USA). Enumerated data were compared using Fisher’s exact test. Continuous data were presented as mean ± standard deviation and compared using two-sample *t*-tests. Variance analysis was used for the comparisons of data before and after treatment. All statistical tests were two-sided; p < 0.05 was considered to indicate statistical significance.

## Results

### Duration of mechanical ventilation, ICU LOS, fluid balance, and patient survival

The duration of mechanical ventilation and the ICU LOS of patients were significantly lower in the EVLW group than in the PAWP group (*t* = 2.283 and *t* = 2.612, respectively; p *<* 0.05 for both). The 7 d cumulative fluid balance was -783 ± 391 ml in the EVLW group and -256 ± 514 ml in the PAWP group (p < 0.05). Nevertheless, no significant differences were observed in the number of survivors at 28 and 60 d after treatment between the two groups (p = 0.542 for both; Table 2).

**Table 2:** Comparison of duration of mechanical ventilation, ICU LOS and survivals at 28 days and 60 days in the two groups

**Groups**	**Duration of mechanical ventilation (days)**	**ICU length of stay (days)**	**Survival at 28 days (%)**	**Survival at 60 days (%)**
EVLW group	10.13 ± 3.02*	12.53 ± 3.50*	73.33	73.33
PAWP group	12.64 ± 2.89	15.50 ± 2.50	78.57	78.57

Data are shown as mean ± SD, *p < 0.05 versus PAWP values.

### Acute lung injury scores

After treatment with EVLW or PAWP, the acute lung injury scores of the patients gradually decreased. This result differed significantly at 1, 3, and 5 d after treatment (p < 0.05). However, the scores between the two treatment groups were not significantly different (*F* = 1.302, p = 0.273; Table 3).

**Table 3:** Comparison of acute lung injury score in the two groups

**Groups**	**Prior treatment**	**The first day post-treatment**	**The third day post-treatment**	**The fifth day post-treatment**	***F***	**p**
EVLW group	2.783 ± 0.452	2.667 ± 0.654*	2.311 ± 0.526*	1.867 ± 0.516*	8.678	0.000
PAWP group	2.826 ± 0.526	2.738 ± 0.587*	2.381 ± 0.582*	2.092 ± 0.546*	5.092	0.003
*t*	0.237	0.307	0.340	1.141	1.302	0.273
p	0.815	0.761	0.736	0.264		

Data are shown as mean ± SD, *p < 0.05 versus prior treatment.

### Oxygenation index

The oxygenation index dramatically enhanced in the EVLW and PAWP groups as the treatment was continued (p < 0.05 for both groups). Significant differences were observed between the two groups (*F* = 6.437, p = 0.006; Table 4).

**Table 4:** Comparison of oxygenation index in the two groups

**Groups**	**Prior treatment**	**The first day post- treatment**	**The third day post- treatment**	**The fifth day post- treatment**	**The fifth day post- treatment**	***F***	**p**
EVLW group	127.568 ± 18.592	147.066 ± 21.339*	173.266 ± 23.251*^△^	191.333 ± 21.053*^△^	210.066 ± 26.547*^△^	33.118	0.000
PAWP group	133.733 ± 22.917	156.000 ± 26.94*	157.857 ± 21.565*	174.571 ± 16.232*	185.571 ± 17.261*	12.024	0.000
*t*	0.798	0.996	-1.847	-2.388	-2.922	6.347	0.006
p	0.431	0.329	0.076	0.024	0.007		

Data are shown as mean ± SD, *p < 0.05 versus prior treatment. ^△^p < 0.05 versus PAWP group.

## Discussion

ARDS usually occurs with noncardiogenic diseases, such as severe infection, shock, trauma, and burns. ARDS is caused by pulmonary edema in the interstitial and alveolar cells, which can be attributed to the damage of pulmonary capillary and alveolar epithelial cells. Pathophysiologically, increased capillary permeability impairs fluid removal from the alveolar space. The accumulation of protein-rich fluids inside the alveoli severely reduces lung volume and lung compliance, as well as disturbs the ventilation-perfusion ratio. ARDS becomes apparent with progressive hypoxemia, clinical respiratory distress, and the heterogeneity of exudative process in chest radiography [10].

To date, fluid management in patients with ARDS remains an intractable problem. Venous fluid infusion is important for patients with severely damaged pulmonary ventilation to maintain a suitable inner capacity and, consequently, to stabilize blood dynamics and infusion into major organs. However, excess fluid would worsen pulmonary edema and thus damage gas exchange. The strategies used in ICUs for fluid management are classified into two categories: 1) the opening fluid management strategy or wet approach and 2) the restricted or conserved fluid management strategy or dry approach [11]. The ARDS network showed that mortality is not significantly different between the restricted and unrestricted fluid management strategies. However, patients with restricted fluid management (i.e., treatment with diuretics and limited fluid infusion) experience a negative fluid balance on the first week (-136 ± 491 ml vs. 6992 ± 502 ml for unrestricted). Compared with unrestricted fluid management, restricted fluid management yields improved oxygenation index, significantly reduced lung injury score, and significantly shorter ICU LOS [12]. Given the premise of maintaining a stable cycle and organ perfusion, the use of restricted fluid management is more favorable for ARDS patients.

The methods for extenuating pulmonary edema may notably reduce the cardiac output and organ filling defect; however, the incidence of dysfunction in other organs is increased [2,13]. In the present study, the incidence of shock and hypotension did not increase, even when restricted fluid management was used in combination with treatment using diuretics or CRRT. This result may be attributed to our small sample size. Further prospective clinical studies should be performed.

EVLW is a unique method for quantitatively monitoring the injury and permeability of pulmonary capillaries. This parameter is considered an independent risk factor to predict the severity and prognosis of critically ill patients [8,9,14-16]. During the progression of ARDS, the pneumonic oxygenation index and static compliance sharply decrease as EVLW increases [8,17]. Furthermore, CVP and PAWP cannot accurately reflect the changes in pulmonary edema during an illness because the data are influenced by the altered permeability of the capillaries and the mechanical ventilation being used [3,4,18,19]. Due to time and objective case constraints, the sample size in this study is small, and there is no significant difference at primary endpoint between two groups. Conditions permitting, the sample size should be enlarged for further investigation.

## Conclusions

The prognosis of ARDS can be influenced by several factors, such as age, primary diseases, and complications due to multiple organ failure or severe sepsis. These factors are closely associated with the high fatality rate of ARDS. Despite the absence of evidence that the negative fluid balance can be used as an independent prognostic factor, the results of this study showed that EVLW for restricted fluid management improves the oxygenation index better than PAWP. Thus, the duration of mechanical ventilation and ICU LOS can be reduced. Although no significant differences in the overall mortality were observed between the two treatments, EVLW has more clinical value than PAWP in terms of fluid management in patients with ARDS.

## Competing interests

The authors declare that they have no competing interests.

## References

[B1] Ware LB Matthay MA The acute respiratory distress syndrome N Engl J Med 2002 342 1334 1349 10793167 10.1056/NEJM200005043421806

[B2] Murphy CV Schramm GE Doherty JA Reichley RM Gajic O Afessa B Micek ST Kollef MH The importance of fluid management in acute lung injury secondary to septic shock Chest 2009 136 102 109 10.1378/chest.08-2706 19318675

[B3] Colmenero M Pérez Villares JM Fernández Sacristán MA Garcia Delgado M FernándezMondéjar E Effect of pulmonary artery pressure on extravascular lung water in an experimental model of acute lung injury Acta Anaesthesiol Scand 2005 49 1449 1455 10.1111/j.1399-6576.2005.00785.x 16223388

[B4] Martin GS Eaton S Mealer M Moss M Extravascular lung water in ill patients with severe sepsis: a prospective cohort study Crit Care Med 2005 9 74 82 10.1186/cc3025 PMC1175916 15774053

[B5] Sakka SG Klein M Reinhart K Meier-Hellmann A Prognostic value of extravascular lung water in critically ill patients Chest 2002 122 2080 2086 10.1378/chest.122.6.2080 12475851

[B6] Patroniti N Bellani G Maggioni E Manfio A Marcora B Pesenti A Measurement of pulmonary edema in patients with acute respiratory distress syndrome Crit Care Med 2005 33 2547 2554 10.1097/01.CCM.0000186747.43540.25 16276179

[B7] Phillips CR Chesnutt MS Smith SM Extravascular lung water in sepsis-associated acute respiratory distress syndrome: indexing with predicted body weight improves correlation with severity of illness and survival Crit Care Med 2008 36 69 73 10.1097/01.CCM.0000295314.01232.BE 18090369

[B8] Berkowitz DM Danai PA Eaton S Moss M Martin GS Accurate characterization of extravascular lung water in acute respiratory distress syndrome Crit Care Med 2008 36 1803 1809 10.1097/CCM.0b013e3181743eeb 18496374 PMC2713576

[B9] Craig TR Duffy MJ Shyamsundar M McDowell C McLaughlin B Elborn JS McAuley DF Extravascular lung water indexed to predicted body weight is a novel predictor of intensive care unit mortality in patients with acute lung injury Crit Care Med 2010 38 114 120 10.1097/CCM.0b013e3181b43050 19789451

[B10] Bernard GR Artigas A Brigham KL Carlet J Falke K Hudson L Lamy M Legall JR Morris A Spragg R The American-European consensus conference on ARDS: definitions, mechanisms, relevant outcomes, and clinical trial coordination Am J Respir Crit Care Med 1994 149 818 824 10.1164/ajrccm.149.3.7509706 7509706

[B11] Fernández-Mondéjar E Guerrero-López F Colmenero M How important is the measurement of extravascular lung water? Curr Opin Crit Care 2007 13 79 83 10.1097/MCC.0b013e328011459b 17198053

[B12] Wiedemann HP Wheeler AP Bernard GR Thompson BT Hayden D de Boisblanc B Connors AF Jr Hite RD Harabin AL Comparison of two fluid-management strategies in acute lung injury N Engl J Med 2006 354 2564 2575 16714767 10.1056/NEJMoa062200

[B13] Rizvi K Deboisblanc BP Truwit JD Dhillon G Arroliga A Fuchs BD Guntupalli KK Hite D Hayden D Effect of airway pressure display on interobserver agreement in the assessment of vascular pressures in patients with acute lung injury and acute respiratory distress syndrome Crit Care Med 2005 33 98 103 10.1097/01.CCM.0000150650.70142.E9 15644654

[B14] Kuzkov VV Kirov MY Sovershaev MA Kuklin VN Suborov EV Waerhaug K Bjertnaes LJ Extravascular lung water determined with single transpulmonarythermodilution correlates with the severity of sepsis-induced acute lung injury Crit Care Med 2006 34 1647 1653 10.1097/01.CCM.0000218817.24208.2E 16625129

[B15] Berkowitz DM Martin G Extravascular lung water measurement in acute respiratory distress syndrome Crit Care Med 2009 37 378 19318817 10.1097/CCM.0b013e31818f292f

[B16] LeTourneau JL Pinney J Phillips CR Extravascular lung water predicts progression to acute lung injury in patients with increased risk Crit Care Med 2012 40 847 854 10.1097/CCM.0b013e318236f60e 22036857 PMC3272541

[B17] Easley RB Mulreany DG Lancaster CT Custer JW Fernandez-Bustamante A Colantuoni E Simon BA Redistribution of pulmonary blood flow impacts thermodilution-based extravascular lung water measurements in a model of acute lung injury Anesthesiology 2009 111 1065 1074 10.1097/ALN.0b013e3181bc99cf 19809280 PMC2805407

[B18] Boussat S Jacques T Levy B Laurent E Gache A Capellier G Neidhardt A Intravascular volume monitoring and extravascular lung water ill septic patients with pulmonary edema Intensive Care Med 2002 28 712 718 10.1007/s00134-002-1286-6 12107676

[B19] Hudson E Beale R Lung water and blood volume measurements in critically ill Curr Opin Crit Care 2000 6 222 226 10.1097/00075198-200006000-00014

